# Microbial production and chemical transformation of poly-γ-glutamate

**DOI:** 10.1111/1751-7915.12072

**Published:** 2013-07-15

**Authors:** Makoto Ashiuchi

**Affiliations:** Agricultural Science, Graduate School of Integrated Arts and Sciences, Kochi UniversityNankoku, Kochi, 783-8502, Japan

## Abstract

Poly-γ-glutamate (PGA), a novel polyamide material with industrial applications, possesses a nylon-like backbone, is structurally similar to polyacrylic acid, is biodegradable and is safe for human consumption. PGA is frequently found in the mucilage of *natto*, a Japanese traditional fermented food. To date, three different types of PGA, namely a homo polymer of d-glutamate (D-PGA), a homo polymer of l-glutamate (L-PGA), and a random copolymer consisting of d- and l-glutamate (DL-PGA), are known. This review will detail the occurrence and physiology of PGA. The proposed reaction mechanism of PGA synthesis including its localization and the structure of the involved enzyme, PGA synthetase, are described. The occurrence of multiple carboxyl residues in PGA likely plays a role in its relative unsuitability for the development of bio-nylon plastics and thus, establishment of an efficient PGA-reforming strategy is of great importance. Aside from the potential applications of PGA proposed to date, a new technique for chemical transformation of PGA is also discussed. Finally, some techniques for PGA and its derivatives in advanced material technology are presented.

## Introduction

Most plastics and synthetic polymers, such as nylons and acrylic materials, are derived from petrochemicals. These long-lasting polymers are used even for short-lived applications leading to a profound influence on the environment. Plastic materials that are improperly disposed of are serious sources of environmental pollution. The elimination of waste plastics is therefore of value in the following disciplines: surgery, health, catering, packing, agriculture, fishing, environmental protection and other technical endeavours. Increased knowledge of the value of the preservation of environmental systems has resulted in a complete change in the production of conventional and non-degradable polymers. The ultimate goal is to produce earth-friendly biodegradable polymers that will contribute to savings in energy and resources, help to curb the greenhouse effect, encourage the development of eco-compatible processes and products, and diversify agriculture for food production.

**Figure 1:**
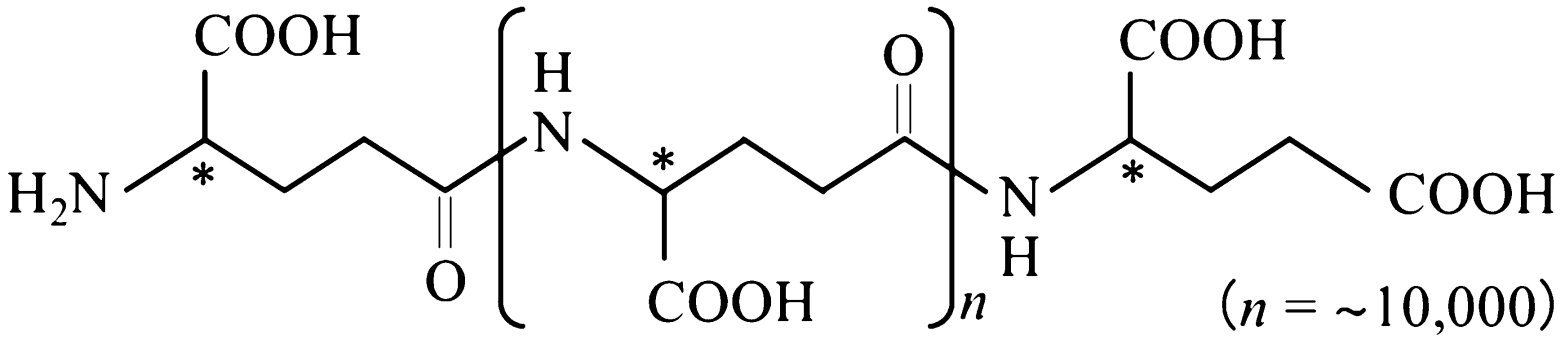
Molecular structure of PGA. The backbone is virtually the same as that of a chemically synthesized (and achiral), high-performance polyamide material, such as nylon-4 (Ashiuchi, 2011). The chirotopic carbon(s) of PGA are usually indicated with asterisk(s).

A biopolymer with a nylon-like backbone and structurally similar to polyacrylic acid, poly-γ-glutamate (PGA; Fig. 1), is attracting commercial interest (Bajaj and Singhal, 2011). If PGA is lyophilized so that its moisture content is < 5% of its total weight, it possesses thermoplastic properties (Ashiuchi *et al*., 2013a), and PGA with multiple chirotopic carbons is fairly biodegradable (Ashiuchi and Misono, 2002). Currently, two distinct PGA-reforming strategies, esterification (Yahata *et al*., 1992) and polymer γ-irradiation techniques (Choi and Kunioka, 1995), have been proposed. Indeed, they may provide plastics and hydrogels; however, the merits in environment and industry (e.g. biodegradability and recyclability) of these newly created polymers cannot be assumed on the basis of those of PGA itself because of the irreversible modification of the PGA chains. Hereafter, it is desired that PGA is transformed into plastics by strong but reversible binding with certain common (and preferably safe) chemicals. PGA should be recovered *via* the liberation of such chemicals from the waste materials. In the study, this idea is tentatively called ‘chemical transformation’ of polymers.

This review focuses on the occurrence and physiology of PGA (as a basis for obtaining a better understanding of its microbial production), polymer synthesis and localization (towards the creation and designing of genetically engineered mass producers of PGA), its potential applications (for bioremediation, functional food ingredients, health care, pharmaceutics and advanced biochemistry), and its chemical transformation (towards the development of feasible bio-nylons).

## Occurrence and physiology

To date, three different types of PGA have been identified (Table 1): the homo polymer of d-glutamate (D-PGA), the homo polymer of l-glutamate (L-PGA), and the random copolymer consisting of d- and l-glutamate (DL-PGA). Current information about the molecular physiology of PGA implies that it functions as an adaptation agent in various environments.

**Table 1:** Biochemical comparisons of some PGA producers

Producers	Molecular masses (kDa)	Stereo-chemistry (d-Ratios; %)	d-Glu-supplying enzyme candidates
Gram-positive bacteria			
*Bacillus subtilis*	10–10000	20–80	GLR, DAT
*Bacillus anthracis*	*n.d.*	100	GLR, DAT
*Bacillus megaterium*	> 1000	5–10	GLR, DAT
*Bacillus halodurans*	< 20	0	GLR, DAT
*Staphylococcus epidermidis*	*n.d.*	40–50	GLR, DAT
Gram-negative bacteria			
*Fusobacterium nucleatum*	*n.d.*	*n.d.*	GLR
Archaea			
*Natrialba aegyptiaca*	> 1000	0	–
*Natronococcus occultus*	< 20	0	–

GLR, glutamate racemase; DAT, d-amino acid aminotransferase; –, absence of any d-glutamate-supplying enzymes. *n.d.*, not determined.

### DL-PGA producers

DL-PGA is found in the mucilage of a Japanese fermented soybean food known as *natto* (Ashiuchi and Misono, 2002). Some strains of *Bacillus subtilis* are traditionally utilized as the *natto* starter (Sawamura, 1913). DL-PGA producers of *Bacillus* are classified into two groups: exogenous glutamate-dependent and -independent groups. *B. subtilis* IFO 3335 (Kunioka and Goto, 1994); MR-141 (Ogawa *et al*., 1997); F-2-01 (Kubota *et al*., 1993); subsp. *chungkookjang* (Ashiuchi *et al*., 2001a) and *Bacillus licheniformis* ATCC 9945A (Thorne *et al*., 1954; Leonard and Housewright, 1963; Cromwick and Gross, 1995; Pérez-Camero *et al*., 1999) are included in the former category. *B. subtilis* 5E (Shih and Van, 2001); TAM-4 (Ito *et al*., 1996) and *B. licheniformis* A35 (Cheng *et al*., 1989); S173 (Kambourova *et al*., 2001) belong to the latter group. *Bacillus* DL-PGA is generally accumulated in culture media ( Table 2) and therefore is considered a long-chain *exo*-polymer with various molecular masses of 250 to 5000 kDa (Park *et al*., 2005).

**Table 2:** Over-accumulation of PGA in media by *B. subtilis* and its related strains (Ashiuchi and Misono, 2002)

Strains	Important components in media	Culture conditions	Yield (g l^−1^)
Glutamate-dependent PGA producers			
*B. subtilis* IFO 3335	L-Glutamate (30 g l^−1^); (NH_4_)_2_SO_4_ (30 g l^−1^); citric acid (20 g l^−1^)	37°C, 2 days	10–20
*B. licheniformis* ATCC 9945A	L-Glutamate (20 g l^−1^); NH_4_Cl (7 g l^−1^); citric acid (12 g l^−1^); CaCl_2_ (0.2 g l^−1^); MnSO_4_^.^7H_2_O (0.3 g l^−1^)	37°C, 2–3 days	35
*B. subtilis* (*natto*) MR-141	L-Glutamate (30 g l^−1^); maltose (60 g l^−1^); soy sauce (70 g l^−1^)	40°C, 3–4 days	35
*B. subtilis* subsp. *chungkookjang*	L-Glutamate (20 g l^−1^); sucrose (50 g l^−1^); NaCl (0.5-5.0 g l^−1^)	30°C, 5 days	13.5–16.5
*B. subtilis* F-2-01	L-Glutamate (70 g l^−1^); glucose (1 g l^−1^); veal infusion broth (20 g l^−1^)	30°C, 2–3 days	50
Glutamate-independent PGA producers			
*B. subtilis* TAM-4	NH_4_Cl (18 g l^−1^); fructose (75 g l^−1^)	30°C, 4 days	20
*B. licheniformis* A35	NH_4_Cl (18 g l^−1^); glucose (75 g l^−1^); MnSO_4_^.^7H_2_O (0.04 g l^−1^); HNO_3_ (20 g l^−1^)	30°C, 3–5 days	8–12
*B. licheniformis* S173	NH_4_Cl (4 g l^−1^); citric acid (20 g l^−1^); Mn^2+^, Fe^2+^, Ca^2+^, Zn^2+^ (1 mM each)	37°C, 30 h	1.27

Other than *Bacillus*, some strains of *Staphylococcus epidermidis* also produce DL-PGA as a capsular polymer for evading mammalian immune defence mechanisms (Kocianova *et al*., 2005).

### D-PGA producer

Microbial PGA production was discovered for the first time in *Bacillus anthracis* (Ashiuchi and Misono, 2002), and, to our knowledge, this is the only sole D-PGA producer among those identified so far (Table 1). Although D-PGA itself is avirulent in mammals, its capsular form completely nullifies the immunity of hosts and eventually promotes severe anthrax symptoms (Keppie *et al*., 1963).

### L-PGA producers

An extremely halophilic archaeon, i.e. *Natrialba aegyptiaca* (Hezayen *et al*., 2001), produces long-chain L-PGA (with molecular masses of more than 1000 kDa) for the prevention of drastic dehydration under extremely high-saline conditions (Ashiuchi and Misono, 2002). Interestingly, salt-inducible PGA (l-glutamate content, ∼95%) was also identified from a halotolerant bacterium, *Bacillus megaterium* (Shimizu *et al*., 2007).

*Bacillus halodurans* (Aono, 1987) and *Natronococcus occultus* (Niemetz *et al*., 1997) produce short-chain L-PGA (with molecular masses of less than 20 kDa) as a secondary cell-wall polymer (e.g. teichuronopeptide) for neutralization of the near-cell surfaces, causing extreme alkalophilicity. Short-chain l-PGA was further identified as the major constituent of sticky substances in the nematocysts of cnidarians (e.g. hydra) as well as the generator (or regulator) of internal osmotic pressure in these organisms (Weber, 1989; 1990).

## Biosynthesis

Some *B. subtilis* PGA producers (Table 2) may have potential industrial application. However, in reality, the polymer productivity and quality may vary dramatically depending on small differences in cultivation factors such as the ionic strength of media, aeration, temperature and culture time. Thus, the establishment of reproducible PGA mass-production techniques is of great urgency.

### Reaction mechanism

**Figure 2:**
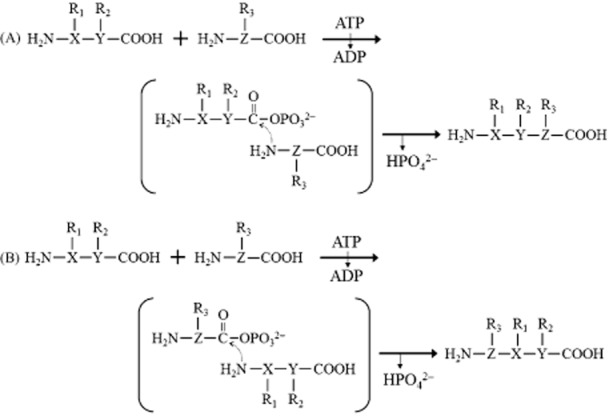
Proposed reaction mechanisms of amide ligases containing either a Rossmann-like fold (A) or ATP-grasp domain (B). In the reaction schemes, X, Y and Z indicate the moieties containing a chirotopic carbon; R_1_, R_2_ and R_3_ are the side chains (*viz.*, amino acid residues). In the case of poly-α-glutamate synthesis, XYZ and R_1,2,3_ are represented as –*CH– and –(CH_2_)_2_–COOH respectively, whereas the former and the latter are altered to –*CH–(CH_2_)_2_– and –COOH in poly-γ-glutamate (PGA) synthesis, for instance.

Elucidation of the mechanism reproducible for the synthesis of PGA would be indispensable for obtaining a better understanding of enzymes involved in its synthesis. Based on the structural features of PGA, such as the introduction of non-proteinaceous d-glutamate and its unusual γ-amide linkage formula, the existence of a novel enzyme that can catalyze non-ribosomal glutamate ligation (viz., polymerization) is predicted. Moreover, the nucleotide formed by coincident ATP hydrolysis is ADP, not AMP (Ashiuchi *et al*., 2001b; Urushibata *et al*., 2002), revealing that PGA is synthesized in an amide-ligation manner (Ashiuchi, 2010). The amide-ligation mechanism is catalyzed by typical amide ligases with a Rossmann-like fold such as murein-biosynthetic enzymes (Eveland *et al*., 1997), or ATP-dependent (ADP-forming) carboxylate-amine/thiol ligases (peptide synthetases) with ATP-grasp domain(s), including glutathione synthetase and d-alanine-d-alanine ligase (Galperin and Koonin, 1997). Both types of amide ligases are commonly characterized by a lack of isomerization activity for amino acid residues in a growing chain, resulting in the substrate having the same stereochemistry as the polymer produced. Therefore, d-amino acid residues in polyamides generated via the amide-ligation mechanism will be derived from free d-amino acids in cells (Ashiuchi *et al*., 2013b). Ashiuchi and colleagues (2004) actually found a membrane-associated DL-PGA synthetic activity from *B. subtilis* subsp. *chungkookjang*, in which both d- and l-glutamate served as direct substrates. Interestingly, there is a noteworthy difference in the proposed catalytic mechanisms of amide ligases (Fig. 2), namely that the Rossmann-type enzymes activate the C-terminal carboxyl residue of the polymers (as the acceptor in peptide elongation; Sheng *et al*., 2000), whereas the ATP-grasp-type enzymes generally phosphorylate the carboxyl group of donor substrates (Fan *et al*., 1995; Kino *et al*., 2009). This may sometimes cause a lack of stereo-exactitude in the former enzymes, resulting in dl-copolymer production. Ashiuchi and colleagues (2001b) previously observed that there was no phosphorylation activity for the monomers of glutamate during the elongation reaction with a *B. subtilis* DL-PGA synthetase, predicting that the enzyme will belong to the superfamily of Rossmann-type amide ligases (Eveland *et al*., 1997).

**Figure 3:**
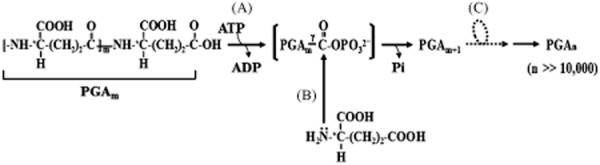
Proposed reaction mechanism for PGA production (Ashiuchi, 2010). Detailed explanation about the reactions steps is described in the Biosynthesis subsection ‘Reaction mechanism’.

The mechanism for DL-PGA synthesis has been proposed (Fig. 3): *step* (A), the transfer of the phosphoryl group of ATP to the C-terminal carboxyl group of a growing chain and the release of the resulting ADP from the active site of the enzyme; *step* (B), the formation of an amide linkage *via* nucleophilic attack of the amino group of either a d- or l-glutamate on the phosphorylated carboxyl group; and *step* (C), the export of DL-PGA after multiple iterations of *steps* (A) and (B) within the enzyme. In principle, PGA is not covalently bound to a membrane-associated enzyme at any stage.

### Molecular enzymology

**Figure 4:**
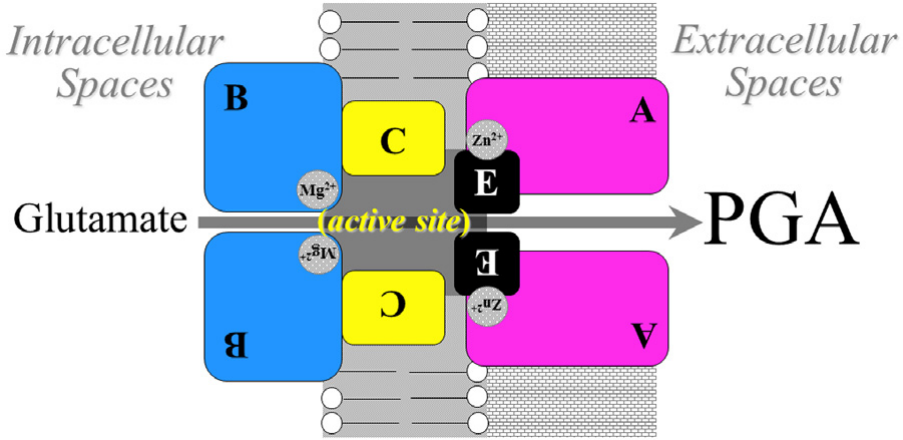
Proposed complex structure of PGA synthetase from *B. subtilis*. All the components of PGA synthetase are essentially membrane associated (Urushibata *et al*., 2002; Ashiuchi, 2010; Ashiuchi *et al*., 2013c).

**Figure 5:**
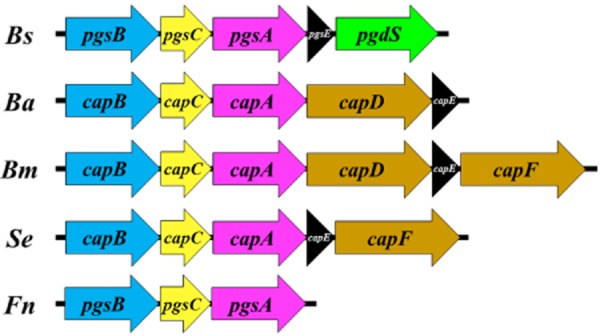
Gene operons for microbial PGA production. Bs, *B. subtilis*; Ba, *B. anthracis*; Bm, *B. megaterium*; Se, *S. epidermidis*; and Fn, *F. nucleatum.* PgsB (CapB) and PgsC (CapC) are structurally similar to a cytosolic enzyme that catalyzes the addition of a short γ-l-glutamyl chain to a folate moiety (FolC) and the *N*-acetyltransferase-domain of *N*-acetylglutamate synthetase respectively. PgsA (CapA) reveals the homology with cytosolic protein serine/threonine phosphatases. PgsE (CapE) is a potent stimulator of PGA production with an assembly accelerator-like structure (Yamashiro *et al*., 2011; Ashiuchi *et al*., 2013c). Detailed information about PgdS and CapD/F is described in the Localization section.

As expected, current research supports the idea that DL-PGA synthetase is a membrane-associated modular protein complex (viz., PgsBCAE) with a Rossmann-type amide ligase-like PgsB component (Fig. 4; Ashiuchi *et al*., 1999; 2001b; Urushibata *et al*., 2002). Research has also been published on the operon organization and molecular machinery involved in D-PGA synthesis in *B. anthracis* (Candela and Fouet, 2006), the L-rich PGA synthesis carried out by *B. megaterium* (DDBJ accession no., AB571872), the DL-PGA synthesis by *S. epidermidis* (Kocianova *et al*., 2005) and the PGA synthesis by *Fusobacterium nucleatum* (Candela *et al*., 2009). All are homologous to the DL-PGA synthesis carried out by *B. subtilis* (Fig. 5). These bacteria possess the responsible genes for d-glutamate synthesis (Kunst *et al*., 1997; Kapatral *et al*., 2002; Read *et al*., 2003; Zhang *et al*., 2003; Eppinger *et al*., 2011) and generally demonstrate the associated enzyme activities in varying amounts (Table 1). In contrast, *N. aegyptiaca*, which is capable of producing long-chain L-PGA, does not contain any pathways for d-glutamate supply (Ashiuchi *et al*., 2013b). *B. halodurans* has the machinery to potentially participate in d-glutamate synthesis similar to other bacilli (Takami *et al*., 2000), though it can produce L-PGA in the absence of d-glutamyl residues. It is noteworthy that neither the *pgs* nor *cap* operon is found in the *B. halodurans* genome (Takami *et al*., 2000), because this strongly implies the participation of an unidentified system: for example, novel L-PGA synthetase(s) with an ATP-grasp domain.

## Localization

**Figure 6:**
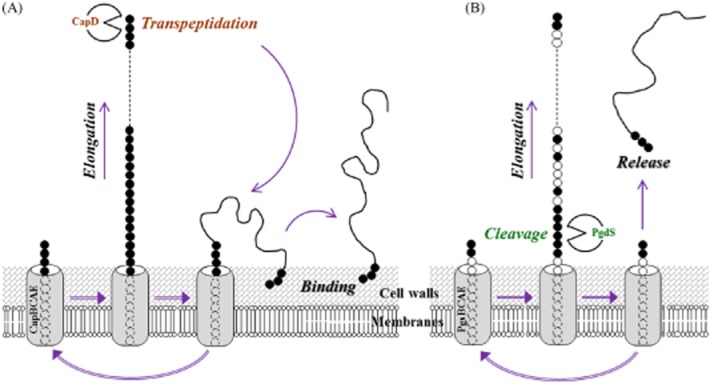
Proposed localization mechanisms of PGA. (A) *B. anthracis* Cap system including CapD enzyme. (B) *B. subtilis* Pgs system with PgdS enzyme. Symbols in elongated polymer structures: *closed circles*, d-glutamyl residues; and *open circles*, l-glutamyl residues of PGA.

PGA is mainly found as a capsular polymer; therefore, its accumulation as an *exo*-polymer, as observed for *B. subtilis* and *B. licheniformis*, the PGA synthetic system of which is virtually the same as that of *B. subtilis* (Wang *et al*., 2011; Yangtse *et al*., 2012), is currently considered a peculiar phenomenon. Comparative genetic analysis of the *pgs* and *cap* operons (Fig. 5) reveals a difference in their downstream genes for PGA cleavage. In fact, *B. anthracis* CapD is essential for the covalent anchoring of D-PGA to peptidoglycans in the cell walls (Fig. 6A; Candela and Fouet, 2006). *B. megaterium* CapD and CapF and *S. epidermidis* CapF structurally resemble the *B. anthracis* enzyme. It was first characterized as an *exo*-type of γ-glutamyltransferase, whereas *B. subtilis* PgdS was identified as an *endo*-type of amidase that catalyzes the γ-glutamyl dd-amidohydrolysis of DL-PGA (Ashiuchi *et al*., 2006). It therefore seems likely that the latter enzyme, different from the former enzyme, has potential to participate in the stimulated release of PGA *in vivo* (Fig. 6B). Further studies on the localization of *F. nucleatum* PGA may be helpful to understanding the function of PgdS enzymes, as any structural gene that corresponds to *pgdS* was absent in the *pgs* operon of *F. nucleatum* (Kapatral *et al*., 2002; *see* Fig. 5). Although the physiological roles of extracellular PGA remain obscure (Ashiuchi and Misono, 2002), Pgs systems should prove more useful as a PGA mass-producer(s) than Cap systems.

## Potential applications

Because PGA, regardless of its stereochemistry, is non-toxic to humans and the environment and is even edible, this chiral-polyamide material is of interest to those in material engineering and related industries. In fact, a wide range of unique applications have been developed (Ashiuchi and Misono, 2002; see Table 3).

**Table 3:** Potential applications of PGA

Categories	Applications	Details
Bioremediation	Flocculants	Substitution for petrochemically synthesized flocculants, such as polyacrylate gels
	Metal absorbents	Removal of heavy metals and radionuclides
Ingredients	Cryoprotectants	Preservation of cryolabile nutrients
	Bitterness-relieving agents	Relief of bitter tastes from amino acids, peptides, quinine, caffeine, and minerals
	Thickeners	Viscosity enhancement for drinks; improvement of food texture; prevention of aging in foods such as bakery products and noodles
	Mineral absorbents	Promotion of absorption of bioavailable minerals, such as Ca^2+^, resulting in increase in egg-shell strength; decrease in body fat of livestock; prevention of human osteoporosis
Health care	Humectants	Use in cosmetic skin-care products
	Dispersants	Uses in detergents, cosmetics, sanitary materials
Pharmaceutics	Drug delivery	Improvement of anticancer drugs; nanoparticle medicines
	Gene vectors	Use for gene therapy
	Curable adhesives	Substitution of fibrin and other synthetic adhesives
Biochemistry	Functional membranes	Separation of metal ions; enantioselection of amino acids
	Extremolytes	Improvement of stability and versatility of macromolecules, enzymes, and bioactive substances

### Bioremediation

Since the Industrial Revolution, we have been releasing into the environment various pollutants such as heavy metals, radionuclides and chemicals that threaten public health and increase the likelihood of a universal shortage of provisions due to profound contamination leading to reduced agricultural output, contaminated water and effects such as acid rain. The remediation of contaminated soils, sediments and waters presents a tremendous challenge, and understanding the interaction of these pollutants with PGA may provide a basis for developing new remediation technologies.

Pötter and colleagues (2001) established a new biological technique that solves serious environmental problems caused by the use of large amounts of liquid manure in intensified agriculture: the reduction of excess NH_3_ in soil and the conversion of the nitrogen into PGA. PGA functions not only as a transit depot for waste nitrogen but also as an earth-friendly fertilizer by which naturally occurring bioavailable cations, such as Fe^2+^, Fe^3+^, Ca^2+^, Zn^2+^, Mg^2+^ and Mn^2+^, can be temporarily condensed and more efficiently transferred to plant rhizospheres (Kinnersley *et al*., 1994).

With the aim of wide adoption in wastewater treatment, dredging and industrial downstream processes, PGA flocculants were developed (Yokoi *et al*., 1995; 1996; Shih *et al*., 2001). In the future, such bio-based flocculants may be used for rapid drinking water purification in addition to downstream processing in food and fermentation industries.

PGA has the potential to be a good absorbent for both bioavailable and toxic cations including rare metals (Ashiuchi and Misono, 2002). Moreover, the biopolymer can bind even an ionic radionuclide, i.e. U^4+^ (He *et al*., 2000), indicating that it may be useful for the removal (or recovery) of heavy metals and radionuclides.

### Functional food ingredients

Current research has highlighted various uses of PGA as a functional food ingredient, for example in cryoprotectants, bitterness-relieving agents, thickeners and mineral absorbents (Shih and Van, 2001; *see* Table 3).

Frequent freezing and thawing cycles cause undesirable deterioration in living cells and bioactive substances, and results in unstable food nutrients. The cyroprotectant properties of PGA (Birrer *et al*., 1994; Mitsuiki *et al*., 1998; Yamasaki *et al*., 2010) makes it suitable for the preservation of cryolabile nutrients.

Bone mass decreases more with increasing aging. Osteoporosis, a significant condition affecting mostly elderly women, arises by a dramatic deterioration of bone density (Price, 1985). Tanimoto and colleagues (2001) found that PGA increased Ca^2+^ solubility *in vitro* and *in vivo*, resulting in intestinal Ca^2+^ absorption. Functional foods supplemented by a proper quantity of PGA may therefore serve as a therapeutic tool for osteoporosis treatment.

### Health care

Based on the fact that PGA has potential applications in water absorption and surface adhesion, some high-performance humectants and dispersants have been developed (Shih and Van, 2001) and utilized in the cosmetic and sanitary industries.

### Pharmaceutics

Many drugs, despite showing great potential as chemotherapeutic agents against human malignancies, have been difficult to use in clinical settings due to their water insolubility (Rowinsky and Donehower, 1995). However, the repurposing of some anticancer drugs is likely to be accomplished *via* the conjugation to water-soluble materials such as short-chain PGAs to the drug (Kishida *et al*., 1998; Kim *et al*., 1999; Li *et al*., 1999; Shih and Van, 2001).

PGA is not only considered to be useful as a vector for gene therapy (Dekie *et al*., 2000), but as a main component of nanoparticle drugs (Akagi *et al*., 2007; Prencipe *et al*., 2009).

To create high-performance adhesives applicable for use in humans, PGA glues have been proposed (Otani *et al*., 1998; Sekine *et al*., 2000). In fact, their mechanical properties were demonstrated to be superior to those of available glues made from fibrins of human blood (Spotniz, 2012).

### Advanced biochemistry

PGA is likely to contribute to the development of advanced biochemical technologies. In the design of functional membranes for metal separation (Bhattacharyya *et al*., 1998) and for enantioselection of amino acids (Lee and Frank, 2002), chemically synthesized poly-α-glutamate (α-PGA) has been employed as a surface-modified material. Hereafter, naturally occurring PGA may replace α-PGA, as the former has significant advantages over the latter in terms of production cost and output, structural features (e.g. molecular size), and environmental impact (e.g. biodegradability).

Recent literature revealed the extremolyte-like functionality of PGA (Yamasaki *et al*., 2010), implying that PGA-coated enzymes may be useful even under extreme conditions where enzymes are usually inactivated: high salt concentrations, dried milieu, extremely low temperatures and high pH. In the near future, PGA may be also used for the processing of conditionally labile macromolecules including DNA and polysaccharides (Tachaboonyakiat *et al*., 2000).

## Chemical transformation

Most of today's plastics are only minimally degraded in nature, and some of the raw materials are harmful to the human body (Dearfield and Abermathy, 1988). To make matters worse, the incineration of waste plastics often generates various endocrine disrupters such as dioxins. Hence, the development of environmentally friendly bio-based plastics is urgently required.

PGA is promising as a novel nylon (polyamide) plastic, although it is not a thermoplastic under ambient humidity. In fact, attempts to use it for industrial purposes for producing important water-insoluble materials such as plastics, fibers and films have been largely unsuccessful because of its hygroscopic nature (Ashiuchi *et al*., 2013a), and the fact that the occurrence of multiple carboxyl residues in PGA makes its plasticization difficult (Ashiuchi and Misono, 2002). Thus, it is necessary to design a method that is effective at transforming the structure and function of the PGA carboxyl so that it can be converted to a water-insoluble bio-nylon material.

### Plasticization and nanofabrication of PGA

**Figure 7:**
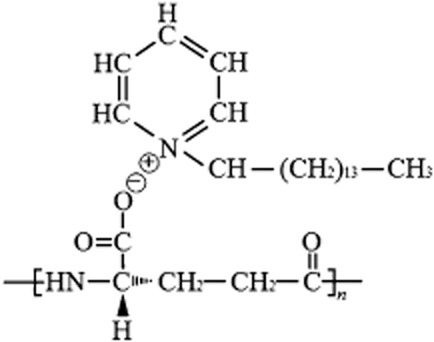
Predicted molecular structure of PGAIC comprised of L-PGA and HDP^+^ (Ashiuchi *et al*., 2013a).

Ashiuchi and colleagues (2013a) recently demonstrated that a compound used in toothpaste, called hexadecylpyridinium cation (HDP^+^), serves as a potent candidate to suppress the extreme hydrophilicity of PGA. In fact, a water-insoluble complex was readily formed by mixing PGA and HDP^+^ at 60°C for 30 min. The nuclear magnetic resonance analysis revealed that it is a stoichiometric ion-complex containing equal number of the carboxyl groups of PGA and HDP^+^ (Fig. 7). This complex is currently called PGAIC. The calorimetric assay of PGAIC implied that it possesses the potential to form a thermoplastic, which could be easily processed into a variety of the shapes and sizes *via* a simple pressurization. Although PGAIC is an ionic complex, it was unexpectedly stable to chemicals such as salts, acids and alkalis, suggesting the involvement of a driving force in the solidification of PGAIC other than a typical ionic interaction. It is noteworthy that PGAIC exhibits good solubility in alcohols (Ashiuchi *et al*., 2013a), whereas PGA itself never dissolves in alcohols. These transformable properties must be taken into account when processing PGA for diverse applications.

Ashiuchi and colleagues (2013a) also succeeded in synthesizing a stable PGA-based nanofiber without a covalent crosslinking (Wang *et al*., 2012), but by using only an ethanol solution of PGAIC. The use of cationic surfactants (e.g. HDP^+^) is likely to provide a promising strategy for fabricating water-friendly anionic polymers (e.g. PGA).

### Antimicrobial performance of plasticized PGA material, PGAIC

The plasticized PGA material, PGAIC, strongly suppressed the proliferation of Gram-negative (*Escherichia coli*), Gram-positive (*B. subtilis*), pathogenic (*Salmonella typhimurium*; *Staphylococcus aureus*) and eukaryotic (*Saccharomyces cerevisiae*) microorganisms. Its antifungal activity was also demonstrated against a prevalent species of Candida (*Candida albicans*) and a filamentous fungus (*Aspergillus niger*). The minimal inhibitory concentrations for fungi were about 0.25 mg ml^−1^; PGAIC is thus classified as potent anti-Candida agent (Aligiannis *et al*., 2001). Owing to the potential of PGA and its derivatives as surface-contact adhesives (Ashiuchi and Misono, 2002), PGAIC-coated polyethylene terephthalate (PET) films have been efficiently manufactured. As a result, zones of growth inhibition appeared when PGAIC-coated PETs were placed in culture plates, whereas PET itself had very little effect on fungal growth. Accordingly, solubilized materials of PGAIC show promise as an antimicrobial material and as a coating substrate.

HDP^+^ is a potent and broadly acting microbicidal agent against bacteria and fungi. The structure of HDP^+^ comprises a hydrophobic chain (aliphatic alkane) and a hydrophilic ring (pyridinium cation). The hydrophobic chain primarily serves to make initial contact with a cell and subsequently is used to attach to membranes, while the hydrophilic ring increases the permeability of the membrane causing the cytoplasmic contents to leak, resulting in cell death (Hugo and Russell, 1982; Petrocci, 1983; Schep *et al*., 1995; Jones *et al*., 1995a,b). PGAIC, however, is formed *via* multiple ionic bonds between the pyridinium cations of the HDP^+^ molecules and the carboxyl anions of PGA (Fig. 7), suggesting that it lacks microbicide functions. Furthermore, the rates of dissociation and diffusion of HDP^+^ from stable PGAIC are limited or very slow, resulting in its resistance to degradation by chemicals. Taken together, these characteristics indicate that PGAIC essentially acts as a microbiostat (non-microbicide).

### Prospects and opportunities

Because antibiotics continue to be used inappropriately, the emergence of drug-resistant strains of fungal pathogens is increasing. Candida species, in particular, cause serious health problems and are often associated with life-threatening mycoses (Calderone, 2002; Lass-Flör, 2009). Therefore, the development of high-performance plastic materials possessing antimicrobial activity as well as biodegradability (or biocompatibility) has become a requisite in the food packaging industries (Liu *et al*., 2009) and for producing advanced pharmaceuticals (Schwartz *et al*., 2012; Song *et al*., 2012). Interestingly, PGAIC has the potential to serve as a bio-plastic material possessing a broad spectrum of antimicrobial activity, resulting from a novel contact-active mechanism of growth inhibition (Ashiuchi *et al*., 2013a). As a class of antimicrobial agents, polymeric materials are generally more efficient and selective (thus safer) than smaller molecules including elemental silver and its composites (Kenawy *et al*., 2007), because they can facilitate prolonged activity owing to the controlled release of toxic moieties from the polymer networks. Long-acting microbiostatic polymers may reduce the spread of drug-resistant microbes.

Over the long term, a biotechnological method for the hyper-elongation and mass-production of useful polyamide materials is needed. Eventually, after optimization, it is hoped that the performance of polyamide materials can be dramatically improved so that they are suitable for widespread industrial applications.
